# Virulence and Antibiotic Resistance of Pathogenic *Aeromonas caviae* from Diseased *Macrobrachium rosenbergii*

**DOI:** 10.3390/microorganisms13061343

**Published:** 2025-06-10

**Authors:** Xinhai Zhu, Qieqi Qian, Anting Chen, Liying Zhou, Yao Zhang, Xiaojian Gao, Qun Jiang, Xiaojun Zhang

**Affiliations:** College of Animal Science and Technology, Yangzhou University, Yangzhou 225009, China; 18761046291@163.com (X.Z.); qieqi2000810@163.com (Q.Q.); chenanting2002@163.com (A.C.); dx120230167@stu.yzu.edu.cn (L.Z.); yao.z@yzu.edu.cn (Y.Z.); gaoxj336@163.com (X.G.); jiangqun1013@163.com (Q.J.)

**Keywords:** *Macrobrachium rosenbergii*, *Aeromonas caviae*, pathogenesis, complete genome

## Abstract

In recent years, viral and bacterial diseases have posed serious challenges to the sustainable development of *Macrobrachium rosenbergii* (giant freshwater prawn) aquaculture, resulting in considerable economic losses across China. Among the bacterial pathogens, *Aeromonas caviae* has emerged as a notable opportunistic agent capable of causing large-scale mortality in various aquatic species. In this study, a highly virulent strain of *A. caviae* (designated GMRS4) was isolated from diseased *M. rosenbergii* exhibiting mass mortality in Yangzhou, Jiangsu Province. The isolate, a Gram-negative bacillus, was identified as the causative agent based on morphological, molecular, and histopathological analyses. Pathogenicity was confirmed through experimental infection, with the strain displaying marked virulence, evidenced by an LD_50_ of 1.91 × 10^6^ CFU/mL at 96 h. Whole-genome sequencing of GMRS4 revealed 4078 coding sequences, including a suite of virulence-associated genes encoding extracellular enzymes (DNase, hemolysin, caseinase, and lecithinase) and toxins (serine protease, elastase, and flagellin). Antimicrobial susceptibility testing indicated resistance to several antibiotics, particularly those in the penicillin and sulfonamide classes, while maintaining sensitivity to quinolones. Genomic analysis further revealed multiple antibiotic resistance genes and virulence genes, offering insights into the pathogenic mechanisms and resistance profile of the strain. This study underscores the threat posed by *A. caviae* to freshwater prawn aquaculture and provides a genetic basis for developing targeted disease management strategies.

## 1. Introduction

As a commercially valuable aquaculture species, *Macrobrachium rosenbergii*, is one of the most important freshwater cultured crustacean species in Asia, with a high annual production of 177,836 tons in 2023 in China (China Fishery Statistical Yearbook, 2024). Such a significant increase in breeding output is primarily due to improvements in seedling and changes in aquaculture models [1,2]. High-density culture is currently the primary farming model of *M. rosenbergii*, resulting in a rising incidence of disease outbreaks caused by bacterial and viral infections [3,4]. Reports indicate that some bacterial pathogens affecting *M. rosenbergii* include *Vibrio* spp. (non-O1/O139 *Vibrio cholerae*, *Vibrio vulnificus*, and *Vibrio alginolyticus*) [5,6,7], *Enterobacter* spp. (*Enterobacter cloacae*) [8], and *Aeromonas* spp. (*Aeromonas sobria* and *Aeromonas veronii*) [9,10]. Generally, outbreaks caused by viral pathogens include infectious precocious virus (IPV) [11], *M. rosenbergii* nodavirus (MrNV) [12], extra small virus (XSV) [13], and decapod iridescent virus 1 (DIV1) [14]. These pathogens cause mass mortalities, significantly diminishing the annual production of cultured *M. rosenbergii* and resulting in significant financial losses for *M. rosenbergii* aquaculture. Therefore, it is crucial to monitor the emergence of pathogens in aquaculture species and to understand their pathogenic mechanisms, as this knowledge can inform the development of effective control measures. This study investigated a mass mortality event of *M. rosenbergii* in Yangzhou, Jiangsu Province. The dominant bacterial strain, GMRS4, isolated from the diseased prawns, was identified as *Aeromonas caviae*.

*A. caviae* is a Gram-negative bacterium belonging to the *Aeromonas* genus and is ubiquitously distributed in various water environments, including freshwater and brackish water [15]. As an opportunistic pathogen, *A. caviae* typically exists harmlessly in the environment or within its host but can cause infections when the host’s immune system is compromised, such as under conditions of stress, overcrowding, or poor water quality [16,17]. In recent years, *A. caviae* has been recognized as an emerging pathogen in aquaculture, causing serious diseases in aquatic animals, such as *Clarias batrachus* [18], *Gelidium pusillum* [19], *Clarias batrachus* [20], *Oreochromis niloticus (Linnaeus, 1758)* [21], *Micropterus salmoides* [22]. Additionally, the extensive application of antibiotics in aquaculture and clinical settings has led to a notable increase in *A. caviae* resistance [23]. Thus, it is imperative to promptly investigate and analyze the pathogenic mechanisms and drug resistance factors of bacterial pathogens to effectively prevent and control this disease in aquaculture.

As is commonly acknowledged, the pathogenic potential of bacteria is largely determined by a range of virulence factors, including colonization, invasiveness, exotoxin, endotoxin, and extracellular enzymes [24]. These factors facilitate the pathogen’s effective colonization, tissue invasion, immune evasion, replication, and pathogenesis in the host [25]. In addition to virulence factors, bacterial survival in hostile environments and resistance to antimicrobial treatment are largely facilitated by resistance mechanisms, such as efflux pumps and β-lactamase production, which are encoded by specific antibiotic resistance genes [26,27]. Unlike single mechanisms, the synergistic interaction of multiple virulence and resistance factors significantly enhances the ability of pathogens to invade the host and survive in the presence of an antimicrobial agent [28]. Consequently, genes encoding both virulence and resistance factors are frequently used to evaluate the pathogenic potential and antimicrobial resistance profiles of bacterial pathogens. Recent advancements in NGS technologies, coupled with the development of sophisticated bioinformatics tools, have significantly enhanced the rapid and precise analysis of entire genomes of various bacterial pathogens. Whole-genome sequencing (WGS) plays a critical role in species identification, gene function annotation, the identification of resistance genes, and the analysis of genomic structural variations, which contribute to understanding the pathogenicity and drug resistance of different pathogens [29,30].

In this study, *A. caviae* GMRS4 was isolated from the hepatopancreas of diseased *M. rosenbergii*, and the pathogenic characteristics of GMRS4 were evaluated through artificial infection, histopathological examination, and analysis of virulence genes. Furthermore, the complete genome of *A. caviae* GMRS4 was sequenced, allowing for the identification of virulence-associated and antibiotic resistance genes to elucidate its pathogenic mechanisms and resistance traits. The present results indicate that *A. caviae* GMRS4 is the causative agent responsible for the mass mortalities observed in *M. rosenbergii* aquaculture, and the genomic analysis will provide insights into the pathogenic mechanisms and antimicrobial resistance of *A. caviae*, which aims to provide a theoretical basis for disease prevention and control in the *M. rosenbergii* industry.

## 2. Materials and Methods

### 2.1. Prawn Disease Description and Pathogen Examination

In August 2023, cultured *M. rosenbergii* (weight: 22.94 ± 0.93 g; length: 128.86 ± 2.48 mm) experienced mass mortality within a few days across several farms in Gaoyou, Jiangsu Province, under a cultural condition of 28 °C in freshwater. The disease has been prevalent for an extended period, with peak outbreaks occurring during the summer, and prawn mortalities reaching approximately 40–50%. Specimens of *M. rosenbergii* exhibiting high mortality rates were obtained from affected farms for pathogen analysis. Bacterial examination was conducted following Gao et al. [31], viral examination was based on Qian et al. [14], and parasites were observed using a light microscope. No viruses or parasites were detected; nevertheless, the primary bacterial strain was isolated from the hepatopancreas and designated as GMRS4. The dominant single colony was subsequently re-streaked onto a Luria–Bertani (LB) agar slant (Hope Bio. Co., Ltd., Qingdao, China), and the purified culture was stored in LB medium supplemented with 30% glycerol at −80 °C for further studies.

### 2.2. Morphological Observation of Aeromonas caviae GMRS4

GMRS4 was cultured in LB broth at 28 °C with shaking at 180 rpm for 18 h. Subsequently, the pure GMRS4 cells were harvested via centrifugation at 6000× *g* for 10 min at 4 °C and washed three times with sterile phosphate-buffered saline (PBS). The bacterial samples were fixed with 0.25 g/L glutaraldehyde at 4 °C and stained with osmium tetroxide (0.1 M dimethylarsinate buffer). The samples were washed with PBS, and excess stain was removed. Subsequently, the samples were gradually dehydrated using a series of ethanol solutions (50%, 70%, 90%, and 100%). After embedding and polymerizing in epoxy resin, the samples were sectioned and placed in a palladium–gold alloy. Flagellated cells were examined using a Zeiss EM10 transmission electron microscope (Zeiss, Oberkochen, Germany) to analyze the types and sizes of the flagella.

### 2.3. Histopathology

Samples from the hepatopancreas, gills, and intestines of naturally infected *M. rosenbergii* were fixed in 10% neutral buffered formalin, dehydrated through an ethanol series, and embedded in paraffin. Subsequently, 6 μm sections were cut and stained with hematoxylin and eosin (H&E) for examination under a light microscope [14]. Tissue samples from healthy prawns were set as a control group.

### 2.4. Identification of Bacterial Isolates

Twenty-two kinds of biochemical reaction tubes (Hangzhou Binhe Microorganism Reagent Co., Ltd., Hangzhou, China) were selected for the biochemical tests, and the phenotypic characteristics of strain GMRS4 were assessed and compared with descriptions in Bergey’s Manual of Systematic Bacteriology [32]. The *16S rRNA* gene of GMRS4 was amplified via PCR using primers specified by Zhang et al. [33]. The amplifications were performed in a 25 μL reaction mixture containing 12.5 μL of Easy Taq PCR Super^®^ Mix (TransGen Biotech, Beijing, China), 0.5 μL of forward and reverse primers (10 mM), 1 μL of DNA template (57.93 ng/μL), and 10.5 μL of ddH_2_O. Following sequencing at Shanghai Sangon Biotech Co., Ltd. (Shanghai, China), the *16S rRNA* sequence homology of GMRS4 was analyzed using BLAST on NCBI. Using the maximum likelihood approach, phylogenetic trees were generated with MEGA 7.0 software [34].

### 2.5. Bacterial Virulence Assay

Healthy *M. rosenbergii* specimens (weight: 2.16 ± 0.32 g) were sourced from a prawn farm in Gaoyou County, Jiangsu Province, China, and reared in a closed-containment aquaculture freshwater system at a constant temperature of 30 °C and dissolved oxygen levels maintained at ≥5 ppm. The prawns were fed twice daily with commercial pellet feed, and water was renewed twice daily, with one-fourth of the total volume replaced each time. After a 7-day acclimation period, the prawns were confirmed to be free of pathogen infections. All animal handling procedures adhered strictly to the guidelines set forth by the Animal Experiment Ethics Committee of Yangzhou University.

For the challenge experiment, strain GMRS4 was incubated in LB broth at 28 °C with continuous shaking at 180 rpm for 18 h. Following incubation, the bacterial culture was serially diluted in sterile PBS (pH 7.4) to achieve final concentrations ranging from 2.4 × 10^8^ to 2.4 × 10^4^ CFU/mL. Prawns were allocated into experimental and control groups, with each group comprising three replicates. In each challenge group, 50 prawns were intraperitoneally injected with 100 μL of bacterial suspension at concentrations of 2.4 × 10^8^, 2.4 × 10^7^, 2.4 × 10^6^, and 2.4 × 10^5^ CFU/mL. Control prawns received 100 μL of sterile PBS. Survival rate was observed daily over a seven-day period, and cumulative mortality data were collected to determine the LD_50_ value of GMRS4 for *M. rosenbergii* using the method of Behreans and Kärber [35].

### 2.6. Determination of Virulence-Related Factors

Exoenzyme activities, including DNase, hemolysin, caseinase, phospholipase, and amylase, were assessed according to Gao et al. [31]. A 5 µL aliquot of bacterial suspension was carefully inoculated onto the center of each plate, followed by incubation at 28 °C for 18 h in triplicate. The presence of extracellular enzyme activity in isolate GMRS4 would be indicated by the formation of a clear lytic halo surrounding the colonies.

The polymerase chain reaction was performed using Easy Taq PCR Super^®^ Mix (Tolo Biotech Co., Ltd., Shanghai, China) to detect the presence of virulence-related genes in the isolate GMRS4, including *ahp*, *ahyB*, *flgM*, *hly*, *alt*, and *aer*. Specific primers were designed based on genomic data (Table 1). PCR conditions were the same as described in Section 2.4. PCR products were analyzed using 1% agarose gel electrophoresis.

### 2.7. Antibiotic Sensitivity Test

To evaluate the antibiotic sensitivity of isolate GMRS4, the Kirby–Bauer disk diffusion method was employed. The bacterial culture was grown overnight and subsequently diluted in sterile saline to reach a final concentration of 1.8 × 10^8^ CFU/mL. A 100 μL aliquot of the prepared bacterial suspension was then evenly distributed across the surface of an LB agar plate. Antibiotic discs were carefully placed at the center of plates, and the plate was placed in an inverted orientation and incubated at 28 °C for a duration of 24 h. Following incubation, the inhibition zone diameters (mm) around each antibiotic disc were recorded and analyzed. The susceptibility of GMRS4 to different antibiotics would be compared following the guidelines provided by Hangzhou Binhe Biological Company.

### 2.8. Whole-Genome Sequencing and Assembly

Genomic DNA was extracted from 1 g of pure bacteria utilizing an optimized SDS-based extraction protocol (Oxford Nanopore Technologies, Oxford, UK). Agarose gel electrophoresis was also used to check the quality of genomic DNA. Following quality assessment of the sample using a NanoDrop One spectrophotometer (NanoDrop Technologies, Wilmington, DE, USA) and a Qubit 3.0 Fluorometer (Life Technologies, Carlsbad, CA, USA), a sequencing library was prepared using the SQK-LSK110 kit (Oxford Nanopore Technologies, Oxford, UK) according to the instructions specified by the manufacturer. Concurrently, a small-fragment library was generated employing the VAHTS^®^ Universal Plus DNA Library Prep Kit for MGI V2/for Illumina V2 (Vazyme, Nanjing, China). After assessing the library quality, sequencing was performed using both the Nanopore PromethION and Illumina NovaSeq 6000 platforms. Raw sequencing reads obtained from the Nanopore system were subjected to quality control, retaining only those with Q scores of 7 or higher and lengths of at least 1600 bp to ensure valid data output. Raw data from second-generation sequencing were filtered using fastp [36], and Unicycler software (https://github.com/rrwick/Unicycler) was utilized to assemble the filtered reads, followed by depth statistics for second-generation sequencing. BWA was used to align Illumina short reads to the assembled genome [37], while the depth statistics for third-generation sequencing were obtained using Minimap2 to align long reads to the assembled genome [38]. Finally, the average sequencing depth for both second- and third-generation sequencing data was calculated using the Samtools depth tool, reflecting the read coverage across different regions [39].

### 2.9. Gene Prediction and Database Annotation

The assembled genome was annotated for coding genes using Prokka [40]. Gene islands present in the genome were predicted using IslandPath [41]. Phage-like sequences in the genome were predicted using the PhiSpy website. The prediction and analysis of genome promoter sequences were conducted utilizing PromPredict software (https://bio.tools/prompredict). To ensure a comprehensive understanding of gene functionality, annotations were carried out based on information from eight key databases, including UniProt [42], KEGG [43], GO [44], Pfam [45], COG [46], TIGRFams [47], RefSeq, and NR. Annotations with an E-value threshold of 1 × 10^−5^ were selected based on the highest sequence similarity. Using the BLAST function in DIAMOND software (https://www.crystalimpact.com/diamond/), sequences were analyzed against the CARD resistance gene database with a stricter E-value threshold of 1 × 10^−10^. BLASTP was used to annotate the target protein sequences based on the VFDB, PHI, ARDB, and CARD databases, and annotations with *E* < 1 × 10^−5^ were selected using DIAMOND software, in order to analyze virulence-associated genes and antibiotic resistance–related genes.

## 3. Results

### 3.1. Electron Microscopic Observation of the Isolate GMRS4

**Figure 1:**
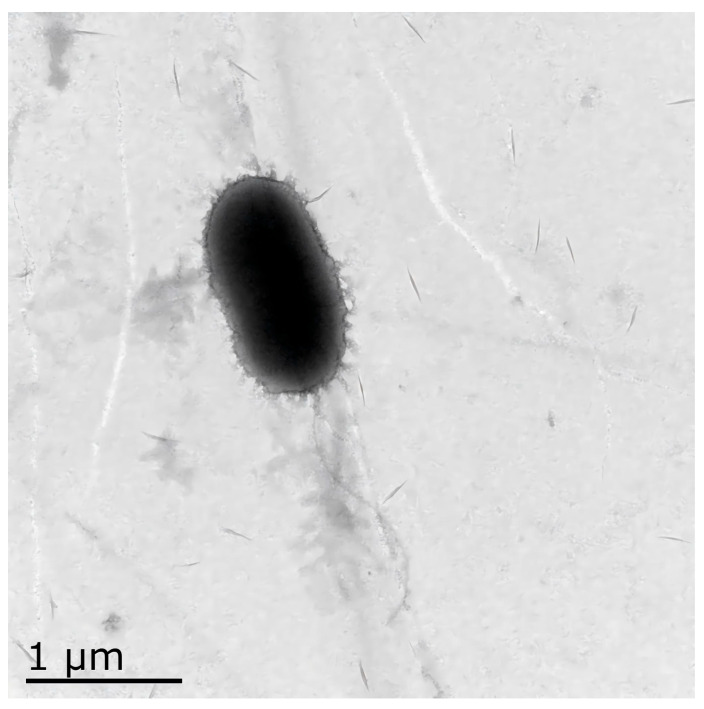
Electron micrograph of GMRS4, scale bar = 0.5 μm. The bacterium appears rod-shaped with blunt, rounded ends and a single polar flagellum. Cell dimension range: length, 1.4–1.8 μm; width, 0.7–1.1 μm.

TEM revealed that GMRS4 was rod-shaped with blunt, rounded ends and possessed a single flagellum. The size of the bacteria ranged from 1.4 to 1.8 μm in length and 0.7 to 1.1 μm in width (Figure 1).

### 3.2. Histological Observation

**Figure 2:**
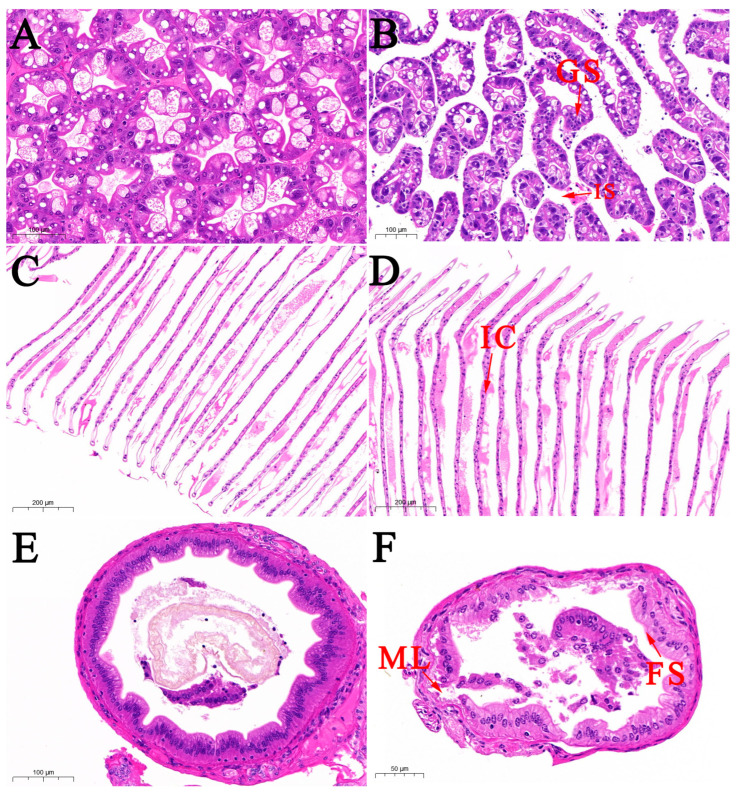
Histological changes in the Hepatopancreas (**A**,**B**), gills (**C**,**D**), and intestines (**E**,**F**) of *M. rosenbergii* infected with isolate GMRS4. GS represents disrupted glandular structure; IS represents enlarged intratubular spaces; IC represents inflammatory cells; FS represents reduced fold structures; ML represents ruptured muscle layer.

Tissue sections showed significant changes in the hepatopancreatic tissue of the diseased prawns. Compared to the control group, the hepatopancreatic tubule lumens and intratubular spaces in naturally infected prawns were notably enlarged, with increased vacuolization of hepatocytes (Figure 2A,B). The glandular structure was disrupted, and the cellular arrangement was disordered, indicating significant damage to the hepatopancreatic tissue. Additionally, a large infiltration of inflammatory cells was observed in the gill tissue of the diseased prawns, and gill cells exhibited swelling, shedding, or necrosis (Figure 2C,D). In the intestinal tissue of naturally infected prawns, the intestinal wall showed large fissures, reduced fold structures, and separation between the columnar epithelium and connective tissue. The muscle layer was ruptured, the connective tissue was atrophied, and there was separation between the muscle and connective tissues, along with disorganization of the villous structures (Figure 2E,F).

### 3.3. Physiological and Biochemical Characterization

The physiological and biochemical traits of isolate GMRS4 are displayed in Table 2. The isolate can utilize cellobiose, xylose, sucrose, rhamnose, mannose, trehalose, aesculin, salicylic acid, glucosamine, arginine dihydrolase, α-methyl-D-glucosamine, and β-galactoside. However, it cannot utilize lactose, arabinose, citrate, myo-inositol, glucuronic acid, melezitose, tartrate, lysine, hydrogen sulfide, or peptone water.

### 3.4. Molecular Identification

**Figure 3:**
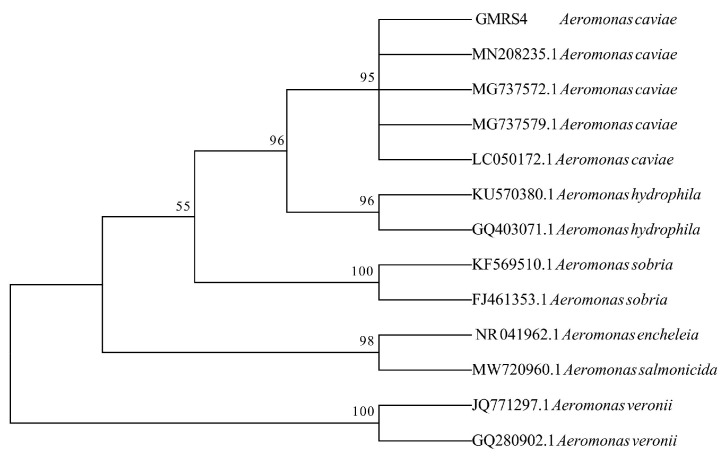
Phylogenetic tree of *Aeromonas* species based on 16S rRNA sequences. Bootstrap values are shown beside the clades. The names of bacteria are indicated beside the accession numbers.

The 16S rRNA gene of GMRS4 was sequenced by Sengon Biotech and assembled using Seqman software (https://www.dnastar.com/software/lasergene/seqman-ngen/) following PCR amplification. Comparison of the 16S rRNA sequence of isolate GMRS4 revealed a 95% sequence identity with *A. caviae* strains (accession number: OM943772.1) in the NCBI Reference RNA Sequences (refseq rna). Phylogenetic analysis further confirmed that isolate GMRS4 is classified as *A. caviae* (Figure 3).

### 3.5. Virulence of A. caviae GMRS4

**Figure 4:**
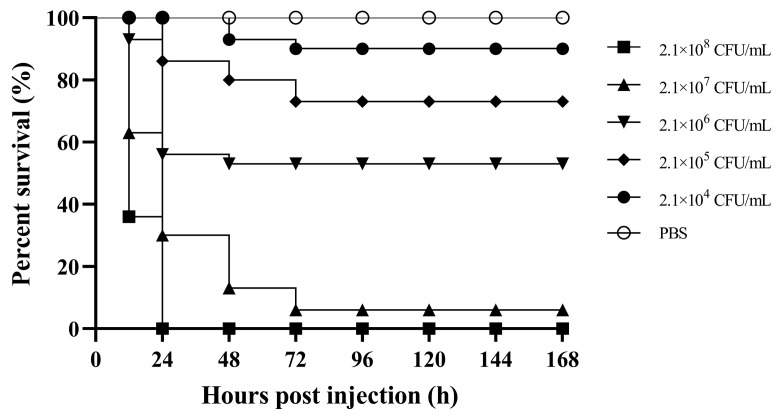
Survival rates of *M. rosenbergii* challenged by GMRS4 at concentrations of 2.1 × 10^8^, 2.1 × 10^7^, 2.1 × 10^6^, 2.1 × 10^5^, and 2.1 × 10^4^ CFU/mL. PBS was set as the control group. Each group included three parallel replicates.

After being infected with different concentrations of *A. caviae* GMRS4, the experimental group of *M. rosenbergii* began to show mortality starting from the first day. Infection with GMRS4 at concentrations of 2.4 × 10^8^, 2.4 × 10^7^, 2.4 × 10^6^, 2.4 × 10^5^, and 2.4 × 10^4^ CFU/mL resulted in 0%, 7%, 53%, 73%, and 90% survival rate at 96 hpi, respectively, while all prawns in the control group survived (Figure 4). Based on these results, the LD_50_ value of *A. caviae* GMRS4 for *M. rosenbergii* was calculated to be 1.91 × 10^6^ CFU/mL at 96 h.

### 3.6. Virulence Factors of GMRS4

**Figure 5:**
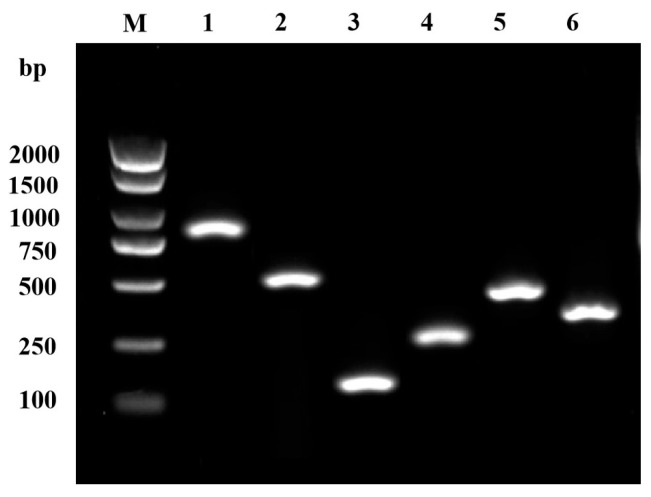
Virulence genes of *A. caviae* GMRS4 via PCR amplification. M, marker; 1, *ahp*; 2, *ahyB*; 3, *flgM*; 4, *hly*; 5, *alt*; 6, *aer*.

**Figure 6:**
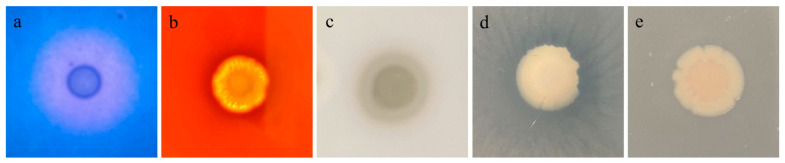
Extracellular enzyme test results of *A. caviae* GMRS4. (**a**) DNase activity; (**b**) hemolysin activity; (**c**) caseinase activity; (**d**) lecithinase activity; (**e**) amylase activity.

PCR analysis was used to identify several virulence-related genes, including serine protease (*ahp*), elastase (*ahyB*), flagellin (*flgM*), hemolysin (*hly*), heat-labile enterotoxin (*alt*), and aerolysin (*aer*). The enzymatic activities of extracellular proteins produced by GMRS4 are presented in Figure 5. GMRS4 exhibits DNase activity, hemolysin activity, caseinase activity, and lecithinase activity, but does not show amylase activity (Figure 6).

### 3.7. Antibiotic Sensitivity of GMRS4

The drug susceptibility of GMRS4 against 35 antibiotics is shown in Table 3. The isolate exhibited low sensitivity to penicillin-class antibiotics, amphenicol-class antibiotics, and sulfonamides but was highly sensitive to quinolone-class antibiotics such as levofloxacin, ofloxacin, and ciprofloxacin. GMRS4 showed moderate sensitivity to macrolide-class and nitrofuran-class antibiotics.

### 3.8. Genome Structure and General Features of A. caviae GMRS4 Genome

**Figure 7:**
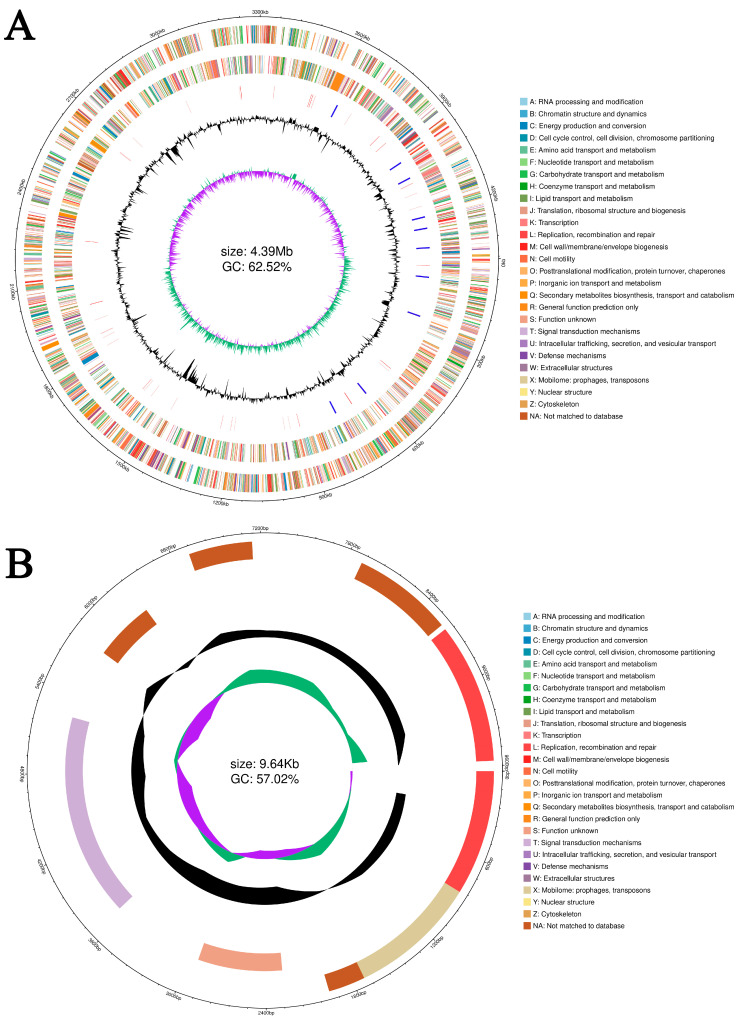
Circular genome map (**A**) and plasmid map (**B**) of *A. caviae* GMRS4. There are coding genes, COG databases, genome size, GC content, and the distribution of GC-skew value from outside to inside.

After quality filtration, the entire genome of *A. caviae* GMRS4 was assembled into one circular chromosome and a circular plasmid (Figure 7). The chromosome of GMRS4 is 4,387,439 bp in length with a G + C content of 62.52%, whereas the plasmid spans 9639 bp with a G + C content of 57% (accession number: PRJNA1247916). In total, 4078 coding genes were identified, accounting for 3,871,119 bp of the genome.

**Figure 8:**
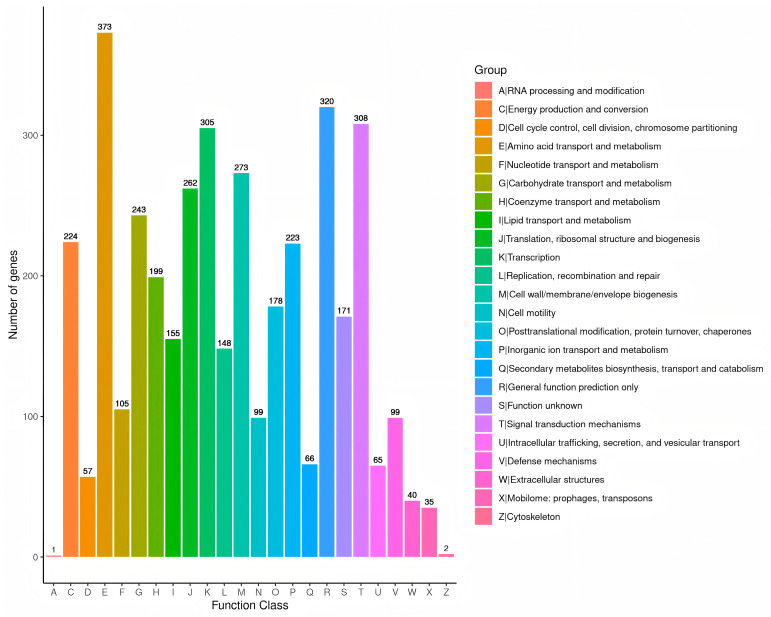
Clusters of Orthologous Gene (COG) functional annotation of the whole genome of *A. caviae* GMRS4. A total of 3951 genes has a COG classification among the 24 categories. The *x*-axis indicates the COG categories, and the *y*-axis indicates the number of genes.

**Figure 9:**
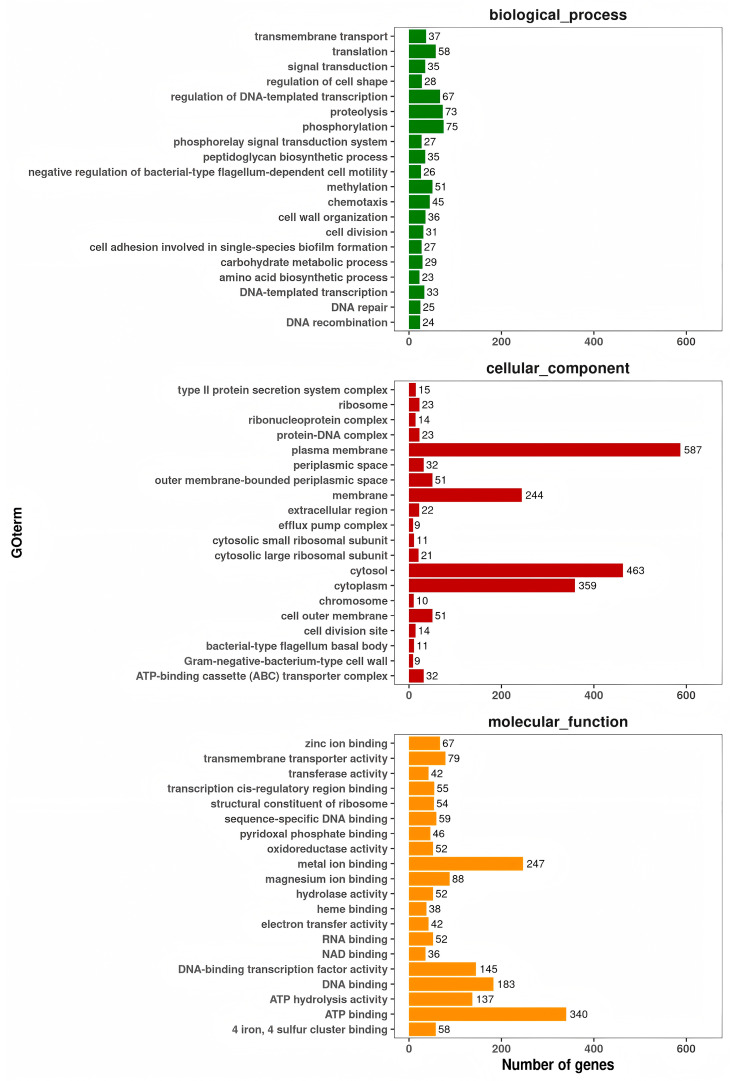
Gene Oncology (GO) functional annotation of the whole genome of *A. caviae* GMRS4. All annotated genes were grouped into 3 functional subcategories: biological process, cellular component, and molecular function.

**Figure 10:**
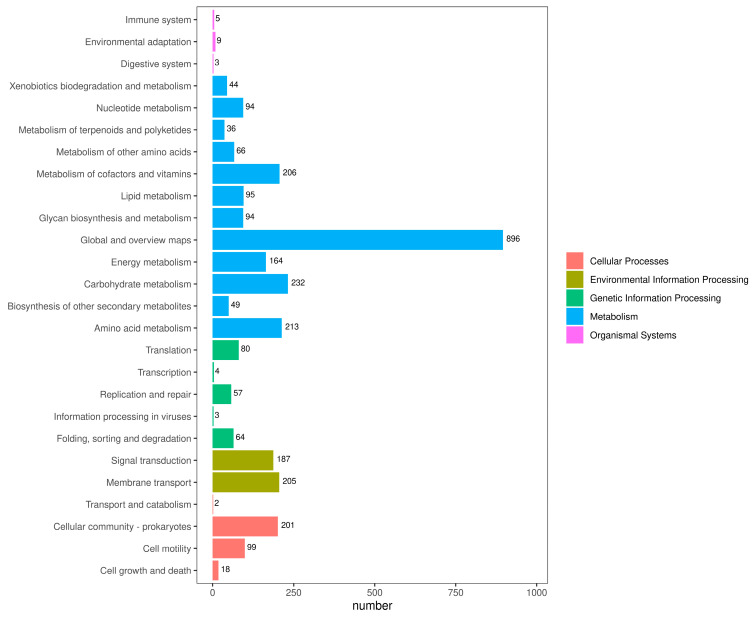
Gene distribution based on Kyoto Encyclopedia of Genes and Genomes (KEGG) classification of *A. caviae* GMRS4. All annotated genes were grouped into 5 functional subcategories: cellular process, environmental information processing, genetic information processing, metabolism, and organismal systems.

In addition, the genome also contained 124 tRNA genes, 33 rRNA genes, and 45 miscRNA genes. The genome of strain GMRS4 contained 379 pseudogenes, 10 CRISPR, and 6 genomic islands. A total of 3951 genes that were annotated according to the COG analysis were divided into 24 functional groups (Figure 8); 4688 genes that were annotated according to the KEGG analysis were divided into five categories and 26 functional groups (Figure 9); and 10,522 genes that were annotated according to GO were divided into three categories and 2445 GO terms (Figure 10).

### 3.9. Prediction of Virulence Genes of A. caviae GMRS4

Analysis compared using the Virulence Factor Database (VFDB) revealed 1184 coding sequences in the GMRS4 genome that are potential virulence genes. Among these, the majority are associated with polar flagella (75 genes, 6.33%), capsules (65 genes, 5.49%), and pyoverdine (47 genes, 3.97%) (Table 4). Additionally, 1806 genes were identified in the PHI database, which may be associated with pathogen–host interactions. Among them, 21 genes were classified as “lethal”, 130 genes were classified as “increased virulence”, 323 genes were classified as “unaffected pathogenicity”, and 1019 genes were classified as “reduced virulence”.

### 3.10. Prediction of Resistance Genes of A. caviae GMRS4

To gain deeper insights into the drug resistance profile of strain GMRS4, the key resistance genes were identified using predictions based on the ARDB and CARD databases. Two resistance genes (baca: ctg_03842; qnrs: ctg_04073) were detected in the ARDB database, which predicts that GMRS4 is resistant to bacitracin and fluoroquinolone (Table 5). Additionally, according to the CARD database annotation, the GMRS4 genome contains eight drug-resistant genes related to fluoroquinolone antibiotic, tetracycline antibiotic, cephalosporin, penam, glycopeptide antibiotic, diaminopyrimidine antibiotic, phenicol antibiotic, and elfamycin antibiotic. The primary function of these genes is to mediate antibiotic resistance through various mechanisms such as antibiotic efflux, antibiotic inactivation, antibiotic target alteration, and antibiotic target protection (Table 6).

## 4. Discussion

As one of the most common pathogenic bacteria in aquaculture, *Aeromonas* species are widely distributed in both freshwater and marine environments and can cause a variety of diseases in aquatic animals, including fish, prawns, and crabs. The most common species, *Aeromonas hydrophila*, infects a variety of aquatic organisms and has been extensively studied to develop therapeutic strategies. It is endemic in several countries and is considered the most frequent *Aeromonas* species in Japan [48]. However, there are few reports on *A. caviae* in aquaculture, and most of them focus on fish. Therefore, it is crucial to study *A. caviae* infection in economically important prawns for developing strategies to combat its spread. In this study, a representative dominant strain, GMRS4, was isolated from diseased *M. rosenbergii*, with severe, serious histopathological signs, including hepatopancreatic tubule lumen and intertubular space expansion, increased hepatocyte vacuolization, severe gill inflammation, and large fissures with reduced fold structures in the intestinal wall. Then, the strain was identified as *A. caviae*. In vivo experimental infection revealed that the LD_50_ of *A. caviae* GMRS4 for *M. rosenbergii* was 1.91 × 10^6^ CFU/mL at 96 h, indicating its high virulence toward this species. Previous research has also confirmed that *A. caviae* is a highly virulent pathogen affecting various aquatic animals. According to Xue et al., *A. caviae* exhibited high virulence toward *M. salmoides*, leading to acute mortality in aquaculture, with an LD_50_ of *A. caviae* WH21406 recorded at 3.46 × 10^5^ CFU/mL [22]. Similarly, Wu et al. reported that *A. caviae* demonstrated β-hemolysis, and the LD_50_ of strain L2 for *C. gibelio* was determined to be 1.33 × 10^6^ CFU/mL [49]. Furthermore, *A. caviae* can infect not only fish but also crustaceans, causing significant mortality. Zhou et al. revealed the strong pathogenicity of *A. caviae* to *Eriocheir sinensis* through experimental infection, with an LD50 value of 1.6 × 10^6^ CFU/mL [50]. Zeng et al. confirmed *A. caviae* as a pathogen of yellow leg disease in *P. vannamei* [51].

The high pathogenicity of bacteria results from a complex interplay of factors that enable them to invade, colonize, and cause disease in the host. These factors are typically categorized into virulence factors, environmental influences, and host factors, with virulence factors playing a key role [52]. The virulence factors of Aeromonas spp. mainly consist of adhesion factors (fimbriae/pili and adhesins), toxins (exotoxins, endotoxins, and hemolysins), extracellular enzymes (proteases, phospholipases, gelatinase, and urease), etc. [53]. In this study, several extracellular enzymes and toxins, including DNase activity, hemolysin activity, caseinase activity, and lecithinase activity, were detected in *A. caviae* GMRS4. By degrading host DNA, DNase helps *Aeromonas* spp. escape immune traps like NETs, facilitate biofilm formation and dispersal, promote tissue damage, acquire nutrients, and even acquire new genetic material through horizontal gene transfer [54]. Caseinase facilitates nutrient acquisition by degrading casein and enhances virulence by promoting tissue invasion, immune evasion, and inflammation (Esteve). By degrading lecithin in host cell membranes, lecithinase disrupts membrane integrity, leading to cell lysis and contributing to tissue damage. This enables *Aeromonas* spp. to penetrate deeper into tissues, promoting the spread of infection. Hemolysins secreted by *Aeromonas* spp. contribute to pathogen damage in aquatic animals by lysing red blood cells, thereby releasing iron and other nutrients that support bacterial growth [55]. This disruption of host cell membranes also triggers inflammation, facilitates bacterial spread, and impairs the host’s immune defense, ultimately leading to systemic infection and tissue damage.

The pathogenesis of *A. caviae* is a complex process depends on multiple factors, including polar flagella, capsule, and pyoverdine. These items were annotated in the *A. caviae* GMRS4 genome. Polar flagella in *A. caviae* are crucial for its virulence, enabling motility that allows the bacteria to reach infection sites and adhere to host tissues. Additionally, flagella facilitate biofilm formation and immune evasion, enhancing bacterial persistence and resistance to host defenses [56]. The capsule of *Aeromonas caviae* plays a critical role in its virulence by preventing phagocytosis, allowing the bacterium to evade the host immune system [57]. Additionally, the capsule aids in biofilm formation, enhancing bacterial persistence on host tissues and providing resistance to both immune defenses and antimicrobial treatments [58]. Pyoverdine, a siderophore synthesized by *A. caviae*, is essential for its virulence, as it facilitates iron acquisition from the host—an essential element for bacterial proliferation and survival [59]. By binding to iron and transporting it into the bacterial cell, pyoverdine facilitates the pathogen’s proliferation in iron-limited environments, such as the host bloodstream or infected tissues, and contributes to the bacterium’s ability to establish and maintain infection [60]. Additionally, pyoverdine may influence the host immune response, promoting inflammation that supports bacterial survival while potentially causing tissue damage.

In aquaculture systems, antibiotics remain one of the most rapid and effective measures for controlling bacterial diseases [61]. However, overuse of antibiotics is a common issue, often resulting in residual antibiotics in both water and sediment. These residues can exert selective pressure, facilitating the emergence and persistence of antibiotic-resistant pathogens [62]. In this study, the GMRS4 strain exhibited a certain degree of antibiotic resistance. In the CARD database annotation, multiple resistance genes were identified, primarily associated with resistance to fluoroquinolones, tetracyclines, cephalosporins, carbapenems, and other antibiotics. These genes are involved in various resistance mechanisms, including antibiotic efflux, antibiotic inactivation, target modification, and target protection, through which bacteria can control intracellular antibiotic levels via complex biochemical pathways or enzymatic degradation of antibiotics [63]. However, in the ARDB database annotation, only two resistance genes, *baca* and *qnrs*, were identified. The drug resistance mechanism associated with the *baca* gene operates through the synthesis of β-lactamase enzymes, which degrade the β-lactam ring in antibiotics, thereby neutralizing their efficacy [64]. Moreover, the mechanism of drug resistance mediated by the *qnrs* resistance gene involves the modification of target enzymes, such as DNA gyrase and topoisomerase IV, preventing the binding of quinolone antibiotics and reducing their efficacy [65]. However, the antimicrobial susceptibility tests revealed that GMRS4 exhibited higher sensitivity to penicillin and lower sensitivity to quinolones. This discrepancy may be attributed to the possible dysfunction of the efflux pump, lack of expression of resistance genes, or enzyme malfunction, which suggests that the presence of antibiotic resistance genes does not necessarily correlate with the manifestation of an antibiotic-resistant phenotype [66].

## 5. Conclusions

In conclusion, this study identified *A. caviae* GMRS4 as a highly virulent pathogen affecting *M. rosenbergii*. Whole-genome sequencing uncovered multiple genes associated with virulence and antibiotic resistance, as annotated in public databases. These findings contribute to a deeper molecular-level understanding of the genetic characteristics, pathogenic potential, antibiotic resistance, and virulence factors of highly pathogenic *A. caviae*.

## Figures and Tables

**Table 1:** The primers used for the PCR.

Sequence ID	Temperature/°C;	Product Length (bp)	Primer Sequences (5′-3′)	Gene
ctg_01684	55	911	ATTGGATCCCTGCCTA	*ahp*
			GCTAAGCTTGCATCCG	
ctg_00796	55	513	ACACGGTCAAGGAGATCAAC	*ahyB*
			ACACGGTCAAGGAGATCAAC	
ctg_01472	63	194	GCTACTGTCAAGCTGGACTC	*flgM*
			AGATTGGCCTCGAAACTG	
ctg_02189	63	289	CGGACGATTATCAGGATGG	*hly*
			CAAGAACGAGTTTCAGTGGC	
ctg_03161	63	442	TGACCCAGTCCTGG	*alt*
			GGTGATCGATCACC	
ctg_04074	63	301	AACCGAACTCTCCAT	*aer*
			CGCCTTGTCCTTGTA	

**Table 2:** Physical and chemical properties of isolate GMRS4.

*Aeromonas caviae* *	GMRS4	Characteristics
+	+	Cellobiose
+	+	Xylose
+	+	Sucrose
+	+	Rhamnose
+	+	Mannose
−	−	Lactose
−	−	Arabinose
+	+	D-Trehalose anhydrous
+	+	Escin
−	−	Citrate
−	−	Inositol
−	−	Dacron
−	−	Raffinose
−	−	Tartrate
+	+	Salicin
−	−	Lysine
−	−	Hydrogen sulfide
+	+	Ammonium Gluconate
+	+	Arginine dihydrolase
+	+	α-Methyl-d-glucose ammonium
−	−	Peptone

Note: “+” indicates positive, “−” indicates negative. “*” refers to data taken from Bergey’s Manual of Systematic Bacteriology.

**Table 3:** Antimicrobial sensitivity of pathogenic *A. caviae* to 35 drugs.

Sensitivity	Diameter of Inhibition Zone/mm	Drug Content of Paper Disc/µg/disc	Drug	Drug Classification
R	8	30	Cefazolin	Cephalosporins
R	8	30	Ceftriaxone	
I	16	30	Cefuroxime	
S	22	75	Cefoperazone	
S	30	30	Cefotaxime	
S	23	30	Ceftriaxone	
S	19	30	Cefepime	
S	20.5	30	Ceftazidime	
S	8	30	Cefoxitin	
S	19	30	Amikacin	Aminoglycosides
S	16	10	Streptomycin	
I	13.5	10	Tobramycin	
S	18	100	Spectinomycin	
I	17	30	Kanamycin	
S	18	10	Gentamicin	
I	13.5	30	Neomycin	
R	8	1	Oxacillin	Penicillin
R	8	10	Penicillin G	
R	8	10	Ampicillin	
R	14	100	Piperacillin	
I	14	15	Erythromycin	Macrolides
I	16	15	Clarithromycin	
R	8	2	Clindamycin	
S	19	30	Tetracycline	Tetracyclines
S	20	30	Minocycline	
S	26	10	Norfloxacin	Quinolones
S	24	5	Ciprofloxacin	
S	23	5	Levofloxacin	
S	25	5	Ofloxacin	
S	26	10	Norfloxacin	Polypeptide
S	24	5	Ciprofloxacin	Furan
S	23	5	Levofloxacin	Amphenicols
S	25	5	Ofloxacin	Monobactams
I	11.5	30	Polymyxin B	Amides
R	8	23.75	Trimethoprim/sulfamethoxazole	Sulfonamides

Note: S sensitive; I intermediate; R resistant.

**Table 4:** VFDB database annotation in the complete genome of *A. caviae* GMRS4.

Number	Related Gene	VF Name	VF Category
65	bcs1, kpsD, cpsA/uppS, lipA, kpsF, etc.	Capsule	Immune modulation
39	lpxC, lpxH, galE, licA, lpxB, msbB, waaQ, etc.	LOS	
11	sadH, fadD13, adhD, etc.	MymA operon	
11	ddrA, ppsC, etc.	PDIM	
27	lpxA/glmU, flmF2, etc.	LPS	
14	YPO_RS16495, YPTB_RS05510, etc.	O-antigen	
15	fbpB, fbpC, etc.	FbpABC	
22	hitC, hitB, hitA, etc.	HitABC	
16	sugC, sugA, sugB, etc.	Trehalose-recycling ABC transporter	
14	hemX, hemD, hemL, hemG, hemN, hemB, etc.	Heme biosynthesis	
11	allB, allS, allR, etc.	Allantion utilization	
47	pvdQ, PA2383, pvdN, pvdE, pvdI, ptxR, etc.	Pyoverdine	
17	pdxJ, CFF8240_RS05385, fleQ, flgP, fliL, etc.	Flagella	Motility
75	flgB, flrC, fliE, flaA, fliK, flgM, flgA, cheY, etc.	Polar flagella	
17	tapV, tapP, ASA_RS18105, tapU, tppF, etc.	Tap type IV pili	Adherence
16	mshN, mshC, mshJ, mshP, mshI1, etc.	MSHA type IV pili	
20	frpC, etc.	RTX protein	
25	rpoN, pilS, pilH, etc.	Type IV pili	
14	vpdC, lirB, LPG_RS00105, lidL, etc.	Dot/Icm T4SS secreted effectors	
14	exeM, exeN, exeK, exeH, exeA, exeB, exeL, etc.	Exe T2SS	
10	ETAE_RS04220, MAFF_RS25805, etc.	T3SS	
12	CBUK_RS04750, coxDFB4, rimP, etc.	T4SS secreted effectors	
14	mucD, algW, mucC, algQ, mucP, algU, etc.	Alginate	
10	bopD, etc.	BopD	
34	papR, etc.	PlcR-PapR quorum sensing	
17	rtxA, etc.	RtxA	Exotoxin
23	acfB, etc.	ACF	

**Table 5:** ARDB database annotation in the complete genome of *A. caviae* GMRS4.

Number	SeqList	Resistance Profile	Description	Resistance Type
1	ctg_03842	bacitracin	Undecaprenyl pyrophosphate phosphatase, which consists in the sequestration of Undecaprenyl pyrophosphate.	baca
1	ctg_04073	fluoroquinolone	Pentapeptide repeat family, which protects DNA gyrase from the inhibition of quinolones.	qnrs

**Table 6:** CARD database annotation in the complete genome of *A. caviae* GMRS4.

AMR Gene Family	Resistance Mechanism	Drug Class	ARO	Best_Hit_ARO	ORF_ID
resistance-nodulation-cell division (RND) antibiotic efflux pump	antibiotic efflux	fluoroquinolone antibiotic; tetracycline antibiotic	3000777	adeF	ctg_01323
resistance-nodulation-cell division (RND) antibiotic efflux pump	antibiotic efflux	fluoroquinolone antibiotic; tetracycline antibiotic	3000777	adeF	ctg_01375
TRU beta-lactamase	antibiotic inactivation	cephalosporin; penam	3004450	TRU-1	ctg_02948
glycopeptide resistance gene cluster; vanT	antibiotic target alteration	glycopeptide antibiotic	3002972	vanT gene in vanG cluster	ctg_02455
resistance-nodulation-cell division (RND) antibiotic efflux pump	antibiotic efflux	fluoroquinolone antibiotic; diaminopyrimidine antibiotic; phenicol antibiotic	3005069	rsmA	ctg_00475
elfamycin resistant EF-Tu	antibiotic target alteration	elfamycin antibiotic	3003369	Escherichia coli EF-Tu mutants conferring resistance to Pulvomycin	ctg_03757
elfamycin resistant EF-Tu	antibiotic target alteration	elfamycin antibiotic	3003369	Escherichia coli EF-Tu mutants conferring resistance to Pulvomycin	ctg_03776
quinolone resistance protein (qnr)	antibiotic target protection	fluoroquinolone antibiotic	3002791	QnrS2	ctg_04073

## Data Availability

The whole-genome sequencing data of *A. caviae* GMRS4 have been deposited in the NCBI Sequence Read Archive (SRA) under the accession number SRR33018161 (https://trace.ncbi.nlm.nih.gov/Traces?run=SRR33018161, accessed on 28 May 2025).
